# Down-angulation puncture method to avoid vascular injury during endoscopic ultrasound-guided hepaticogastrostomy

**DOI:** 10.1055/a-2686-3278

**Published:** 2025-09-09

**Authors:** Haruo Miwa, Yugo Ishino, Kazuki Endo, Ritsuko Oishi, Yuichi Suzuki, Hiromi Tsuchiya, Shin Maeda

**Affiliations:** 1 26437 Gastroenterological Center, Yokohama City University Medical Center, Yokohama, Japan; 2 Department of Gastroenterology, Yokohama City University Graduate School of Medicine, Yokohama, Japan

Endoscopic ultrasound-guided hepaticogastrostomy (EUS-HGS) is widely performed in patients with biliary obstruction as an alternative to transpapillary biliary drainage 1 2 . Bleeding associated with EUS-HGS is a rare complication; however, it can occur when blood vessels are present along the needle tract 3 4 5 . Adjustment using the elevator becomes difficult once the needle has been advanced into the liver parenchyma. In such cases, applying down-angulation to the echoendoscope after puncturing the liver parenchyma allows the needle tip to safely displace the vessel ( Fig. 1 ). Herein, we report a case in which the “down-angulation puncture method” was effective to avoid vascular injury during EUS-HGS ( Video 1 ).

**Fig. 1:**
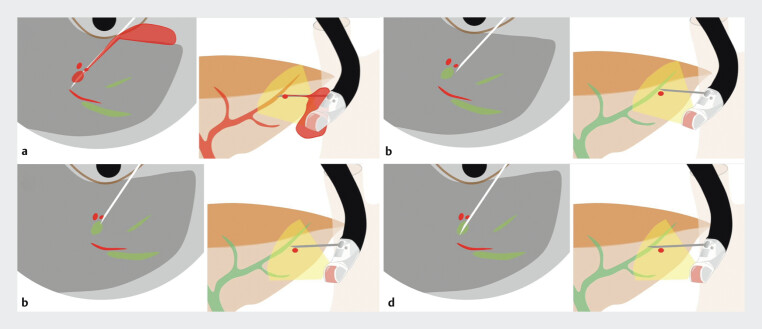
Schema of the down-angulation puncture method **a** Puncture through blood vessels may cause bleeding. **b** A fine needle is inserted, with its tip advanced just to the right of the vessels. **c** Down-angulation of the echoendoscope causes the bile duct to rotate just beneath the needle tip. **d** The bile duct is successfully punctured.

The down-angulation puncture method during EUS-HGS was effective in avoiding vascular injury in the case where blood vessels were present along the needle tract.Video 1

An 81-year-old man with biliary obstruction due to pancreatic cancer underwent EUS-HGS as the initial biliary drainage ( Fig. 2 ). After inserting the echoendoscope (GF UCT 260 Olympus Medical Systems, Tochigi, Japan), multiple dilated intrahepatic bile ducts were visualized from the stomach. Initially, the B2 bile duct branch without blood vessels along the needle tract was selected puncture. Following the puncture, the guidewire was misplaced outside the bile duct due to respiratory movement. For the second attempt, another part of the dilated B2 was selected; however, blood vessels were present along the intended needle tract. A 19-G needle (EZ shot 3 plus; Olympus Medical Systems) was carefully inserted, with its tip advanced just to the right of the artery. Down-angulation of the echoendoscope caused the bile duct to rotate just beneath the needle tip on ultrasound imaging. Subsequently, the bile duct was successfully punctured ( Fig. 3 ). A 0.025-in. guidewire was advanced into the common bile duct. Finally, a partially covered metallic stent was successfully placed ( Fig. 4 ).

**Fig. 2:**
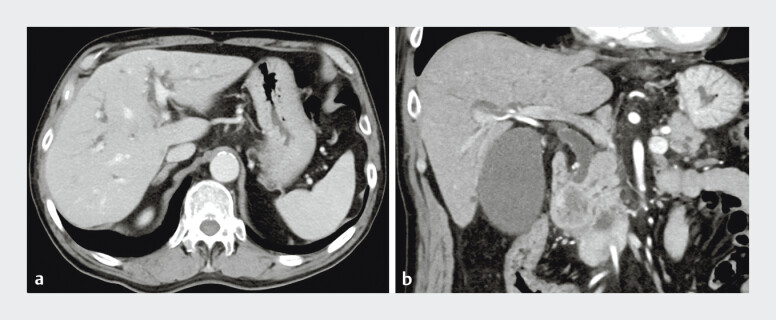
Computed tomography images. **a** Intrahepatic bile ducts in the left lobe are slightly dilated. **b** Distal bile duct is obstructed by pancreatic cancer with duodenal invasion.

**Fig. 3:**
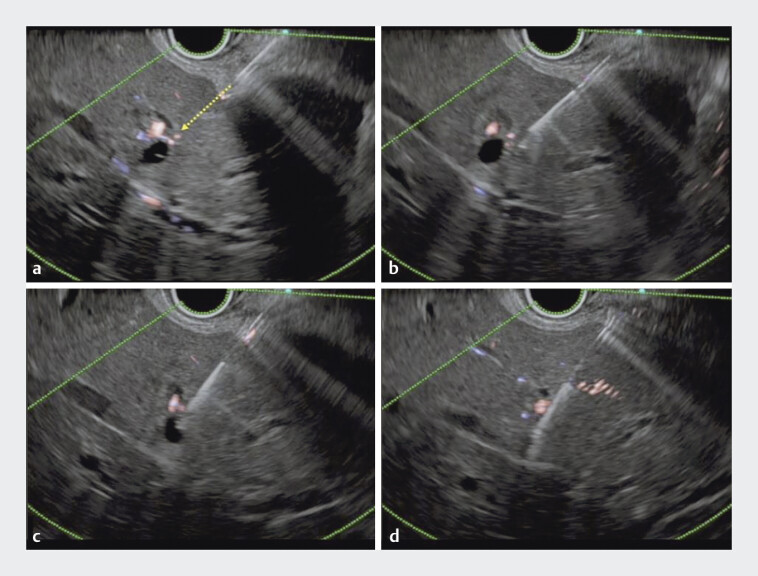
Ultrasonography images of the down-angulation puncture method. **a** Blood vessels are present along the intended needle tract. **b** A 19-G needle is carefully inserted, with its tip advanced just to the right of the vessels. **c** Down-angulation of the echoendoscope causes the bile duct to rotate just beneath the needle tip. **d** Subsequently, the bile duct is successfully punctured.

**Fig. 4:**
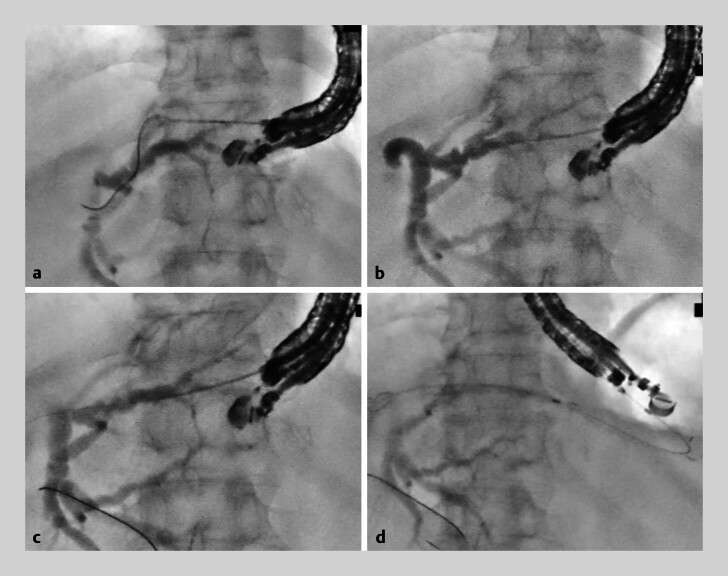
Fluoroscopic images during endoscopic ultrasonography-guided hepaticogastrostomy. **a** Following the first puncture of the B2, the guidewire is misplaced outside the bile duct. **b** B2 branch is successfully punctured in the second attempt using down-angulation puncture method. **c** A guidewire is advanced into the common bile duct. **d** A partially covered metallic stent is successfully placed.

The down-angulation puncture method during EUS-HGS was effective in avoiding vascular injury in the case where blood vessels were present along the needle tract. This technique may enhance the safety and feasibility of the procedure.

Endoscopy_UCTN_Code_TTT_1AS_2AH

## Notes

Endoscopy E-Videos https://eref.thieme.de/e-videos
*E-Videos* is an open access online section of the journal *Endoscopy* , reporting on interesting cases and new techniques in gastroenterological endoscopy. All papers include a high-quality video and are published with a Creative Commons CC-BY license. Endoscopy E-Videos qualify for HINARI discounts and waivers and eligibility is automatically checked during the submission process. We grant 100% waivers to articles whose corresponding authors are based in Group A countries and 50% waivers to those who are based in Group B countries as classified by Research4Life (see: https://www.research4life.org/access/eligibility/ ). This section has its own submission website at https://mc.manuscriptcentral.com/e-videos .
